# Tirzepatide and cardiometabolic parameters in obesity: Summary of current evidence

**DOI:** 10.1111/dom.16549

**Published:** 2025-06-24

**Authors:** Naveed Sattar, Luis‐Emilio García‐Pérez, Angel Rodríguez, Richa Kapoor, Adam Stefanski, Emily R. Hankosky

**Affiliations:** ^1^ School of Cardiovascular and Metabolic Health University of Glasgow Glasgow UK; ^2^ Eli Lilly and Company Indianapolis Indiana USA

**Keywords:** cardiometabolic factors, cardiovascular disease, obesity, tirzepatide

## Abstract

Globally, cardiovascular diseases (CVDs) account for around one‐third of all deaths. Clinical trial evidence suggests that treatment of people with obesity or type 2 diabetes (T2D) and CVD with glucagon‐like peptide‐1 (GLP‐1) receptor agonists reduces the risk of major adverse cardiovascular events, heart failure outcomes and all‐cause mortality. Tirzepatide is a once‐weekly, dual glucose‐dependent insulinotropic polypeptide (GIP) and GLP‐1 receptor agonist that has demonstrated dose‐dependent efficacy in people with obesity, T2D or both, in terms of glycaemic control and bodyweight reduction in clinical trials. This narrative review summarizes the current evidence regarding the effects of tirzepatide treatment on cardiometabolic parameters, including lipid profile, blood pressure and markers of renal function. Additionally, it summarizes the reported impact of tirzepatide treatment on other relevant parameters, such as body composition, liver fat, progression to T2D among individuals with prediabetes, and incidence of heart failure events. Considering the changing landscape of clinical trial evidence of tirzepatide's effects, this review aims to compile the available evidence, which suggests a promising outlook for the cardiometabolic benefits of tirzepatide.

## 1 INTRODUCTION

Cardiovascular diseases (CVDs) present a considerable global public health issue, collectively accounting for one‐third of all deaths in 2019.^1^ According to the American Heart Association, between 2017 and 2020, 48.6% of the United States adult population had some form of CVD (including hypertension).^2^ The economic burden is substantial; direct and indirect costs associated with CVD in the United States between 2019 and 2020 were estimated to be $422.3 billion.^2^

The Semaglutide Effects on Cardiovascular Outcomes in People with Overweight or Obesity trial (SELECT) was the first cardiovascular outcomes trial (CVOT) that provided evidence of a relationship between an approved weight‐reducing therapy (semaglutide 2.4 mg administered subcutaneously once weekly) and atherosclerotic CVD (ASCVD) risk reduction in people living with obesity.^3^ SELECT assessed the impact of semaglutide, a glucagon‐like peptide‐1 (GLP‐1) receptor agonist, on major adverse cardiovascular (MACE) outcomes among people with obesity with preexisting CVD but without diabetes.^3^ Semaglutide treatment significantly reduced the risk of MACE‐3 (non‐fatal myocardial infarction, non‐fatal stroke, cardiovascular death) by 20% compared with placebo (hazard ratio: 0.80, 95% confidence interval [CI]: 0.72 to 0.90; *p* < 0.001), with nominally significant reductions in heart failure outcomes and all‐cause mortality.^3^ The results of this trial have increased interest in obesity management medications in the cardiovascular and other scientific communities. However, there remains a debate on the relative contribution of factors such as weight reduction, improvements in other cardiometabolic parameters (including visceral fat, blood pressure and lipids), as well as other mechanisms, to the reported MACE benefits associated with incretin‐based obesity management medications.^4^

Tirzepatide is a once‐weekly dual glucose‐dependent insulinotropic polypeptide (GIP) and GLP‐1 receptor agonist, approved for the treatment of adults with type 2 diabetes (T2D), for weight management in adults with obesity or overweight with at least one weight‐related comorbid condition, and for obstructive sleep apnoea in adults with obesity.^5^, ^6^, ^7^ Tirzepatide exerts glucose‐lowering effects by activating both GIP and GLP‐1 receptors, potentially in a synergistic or additive manner,^8^ thereby augmenting glucose‐dependent insulin secretion and inhibiting glucagon release. Additionally, tirzepatide transiently delays gastric emptying, which may slow post‐meal glucose absorption and benefit postprandial glycaemia.^9^ Furthermore, by increasing feelings of satiety and fullness, tirzepatide results in decreased food intake and feelings of hunger, leading to appetite regulation and bodyweight reduction.^9^ Tirzepatide also reduces the intensity of food cravings and preferences for high‐calorie foods.^10^ In clinical trials, tirzepatide has demonstrated dose‐dependent efficacy in people with obesity, T2D or both, in terms of glycated haemoglobin (HbA1c) level reduction and bodyweight reduction compared with placebo, GLP‐1 receptor agonists and basal insulin.^11^, ^12^ In this narrative review, we summarize current evidence on the effects of tirzepatide on cardiometabolic parameters. We do so as evidence is changing rapidly, and collating the totality of the effects of tirzepatide on cardiometabolic parameters seen thus far is likely useful to some clinical and research communities.

## 2 EFFECT OF TIRZEPATIDE ON CVD AND CARDIOMETABOLIC PARAMETERS

**FIGURE 1:**
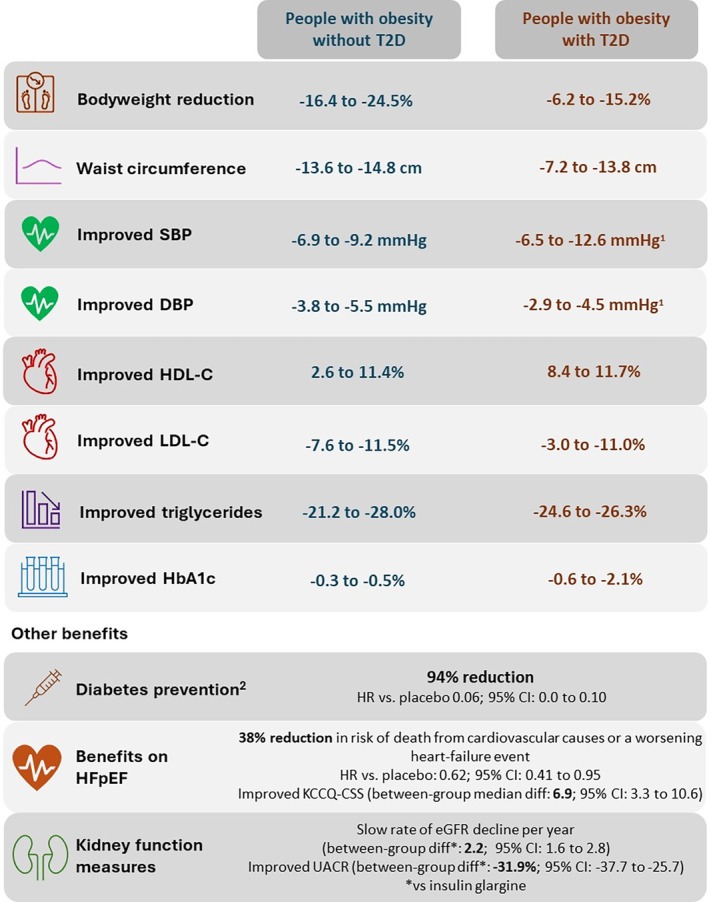
Summary of the treatment effects of tirzepatide relative to comparators on cardiometabolic parameters among people with obesity or overweight with and without T2D: Evidence from randomized clinical trials. Ranges across studies for changes from baseline, or for estimated treatment differences or absolute differences (regardless of efficacy estimand or treatment‐regimen estimand) for tirzepatide (15 mg) versus comparator groups are presented for the cardiometabolic parameters. Please note that ranges may vary depending on the characteristics of the study population and treatment duration. Study‐specific detailed results can be found in Table S1. Results are presented for the efficacy estimand to provide clinicians with insights into what can be expected for patients who stay on treatment. ^1^For people with obesity and T2D, changes from baseline in SBP and DBP values are presented for the treatment‐regimen estimand. ^2^Reduction in risk of T2D in participants with prediabetes in SURMOUNT‐1 was assessed based on glycated haemoglobin and fasting serum glucose levels, and serum glucose levels in a 2‐h oral glucose tolerance test; diagnosis of diabetes was based on ADA guidelines. ADA, American Diabetes Association; CI, confidence interval; DBP, diastolic blood pressure; diff, difference; eGFR, estimated glomerular filtration rate; HbA1c, glycated haemoglobin; HDL‐C, high‐density lipoprotein cholesterol; HR, hazard ratio; KCCQ‐CSS, Kansas City Cardiomyopathy Questionnaire clinical summary score; LDL‐C, low‐density lipoprotein cholesterol; SBP, systolic blood pressure; T2D, type 2 diabetes; UACR, urine albumin–creatinine ratio.

Results from clinical trials have indicated that tirzepatide treatment improves cardiometabolic parameters among people with or without T2D (Figure 1 and Table S1). For example, in SURMOUNT‐1, a phase 3 randomized clinical trial that assessed the safety and efficacy of tirzepatide among people with obesity or overweight without T2D, participants showed significant placebo‐adjusted improvements in systolic blood pressure (SBP), diastolic blood pressure (DBP), triglycerides and waist circumference after 72 weeks of tirzepatide treatment.^13^ Similar improvements in SBP, DBP, high‐density lipoprotein cholesterol (HDL‐C), low‐density lipoprotein cholesterol (LDL‐C) and triglycerides were reported for participants receiving tirzepatide compared with placebo in the phase 3 clinical trials SURMOUNT‐3^14^ and SURMOUNT‐4,^15^ and consistent results were reported for people with T2D in SURMOUNT‐2.^16^ In post hoc analyses of a phase 2 study, treatment with tirzepatide was shown to be associated with reduced circulating levels of biomarkers associated with cardiovascular risk,^17^ in addition to lowered hsCRP concentrations in participants with T2D in SURPASS‐4.^18^

Improvements in cardiometabolic parameters would be predicted to reduce the risk of metabolic and cardiovascular outcomes. A post hoc analysis of the SURMOUNT‐1 trial using a validated risk engine that included modifiable risk factors as model inputs (SBP, total cholesterol, HDL‐C, presence of T2D, blood pressure treatments and current smoking status) showed that following 72 weeks of tirzepatide treatment, the 10‐year predicted risk of ASCVD was significantly reduced compared with placebo.^19^ The predicted relative change in risk of ASCVD from baseline to week 72 ranged from −23.5% to −16.4% for tirzepatide versus 12.7% for placebo.^19^ Specifically, tirzepatide‐treated participants had 2.4 times greater odds (95% CI: 1.7 to 3.5; *p* < 0.001) of improved ASCVD risk profiles from baseline to week 72 than those in the placebo group. The findings of ongoing outcome trials (SURPASS‐CVOT [NCT04255433]^20^ and SURMOUNT‐MMO [NCT05556512]^21^) will provide evidence of the effects of tirzepatide treatment on cardiovascular outcomes.

Increased heart rate has been associated with a higher risk of CVD and mortality.^22^, ^23^ However, despite increases in heart rate, GLP‐1 receptor agonists were not associated with incident arrhythmias and did not increase the risk of cardiac arrhythmias.^24^ A dose‐dependent association was previously demonstrated between tirzepatide treatment and increased heart rate, compared with GLP‐1 receptor agonists and non‐GLP‐1 receptor agonists.^25^ Current evidence, however, does not indicate an adverse association between tirzepatide treatment and cardiovascular events. Rather, the hypothesis is that tirzepatide may lower such outcomes, with the results of ongoing outcome trials expected shortly.

## 3 EFFECTS ON BODY FAT DISTRIBUTION, LIVER FAT AND CHRONIC KIDNEY DISEASE

Sustained bodyweight reduction among people with obesity is associated with decreased cardiometabolic risk, as well as improved insulin sensitivity, pancreatic β‐cell function and hepatic triglycerides.^26^ Recent evidence from the 3‐year SURMOUNT‐1 study demonstrated that participants with prediabetes and obesity or overweight achieved up to 20% bodyweight reduction on average following tirzepatide treatment (15 mg), and maintained it over 3 years with sustained improvements in waist circumference, blood pressure and lipid levels.^27^ As blood pressure and lipids are impacted by weight, these findings further highlight the cardiometabolic benefits of sustained weight reduction.

Additional evidence on the metabolic effects of tirzepatide treatment comes from assessments of changes in body composition and fat distribution. The impact of tirzepatide on body fat distribution was assessed in a subgroup of SURMOUNT‐1 trial participants who underwent dual‐energy x‐ray absorptiometry.^13^ The mean total body fat mass reduction was 33.9% with tirzepatide and 8.2% with placebo (estimated treatment difference [ETD]: −25.7%; 95% CI: −31.4 to −20.0 for the efficacy estimand). The decrease in the ratio of total fat mass to total lean mass was numerically greater with tirzepatide (from 0.93 at baseline to 0.70 at week 72) than with placebo (from 0.95 to 0.88), suggesting an improvement in overall body composition.^13^

While magnetic resonance imaging (MRI) data on body composition and fat distribution are not yet available among tirzepatide‐treated people with obesity without T2D, data from people with T2D might highlight potential benefits of tirzepatide on relevant parameters. The SURPASS‐3 MRI sub‐study characterized changes in liver fat content (LFC), volume of visceral adipose tissue (VAT) and abdominal subcutaneous adipose tissue (ASAT) following 52 weeks of tirzepatide or insulin degludec treatment among a subgroup of SURPASS‐3 participants.^28^ The study reported significant LFC reduction for pooled tirzepatide groups compared with the insulin degludec group (ETD: −4.71%; 95% CI: −6.72 to −2.70; *p* < 0.0001). In addition, tirzepatide treatment significantly reduced the volumes of VAT and ASAT from baseline, while both significantly increased following insulin degludec treatment at 52 weeks. LFC reduction was significantly correlated with baseline LFC and VAT, ASAT and bodyweight reductions in the tirzepatide groups.^28^ Further studies evaluating these outcomes among people with obesity will provide additional insights into the effects of tirzepatide on body composition and fat distribution.

Obesity is an independent risk factor for chronic kidney disease (CKD) and can increase CKD risk by elevating the risk of T2D, hypertension and atherosclerosis.^29^ Weight reduction‐induced mitigation of these risk factors could potentially have a protective effect on obesity‐related CKD.^4^ Preliminary post hoc analyses on surrogate measures of kidney function from SURPASS‐4 suggest that tirzepatide may be associated with improved albuminuria and slow the rate of estimated glomerular filtration rate (eGFR) decline among people with T2D.^30^, ^31^ Furthermore, a post hoc analysis of a pooled population from the SURPASS 1–5 studies showed that tirzepatide was associated with a clinically meaningful decrease in the urine albumin‐to‐creatinine ratio relative to comparators, suggesting potential kidney‐related benefits among people with T2D, with or without CKD.^32^ Additionally, a post hoc analysis of the SURMOUNT‐2 trial demonstrated that among participants with obesity or overweight with T2D and preserved eGFR at baseline, tirzepatide was associated with reduced albuminuria without adversely affecting eGFR.^33^ Further studies are needed to understand the potential association between the effects of tirzepatide on the kidney and its impact on the risk of CVD among people with obesity. TREASURE‐CKD (NCT05536804)^34^ is a randomized, placebo‐controlled study of the effects of tirzepatide on kidney oxygenation and perturbed renal energetics, kidney inflammation, fibrosis and renal blood flow, in participants with overweight or obesity and CKD, with or without T2D. In addition, assessments of kidney function among people with obesity are ongoing for tirzepatide in the SURMOUNT‐MMO (NCT05556512)^21^ trial.

## 4 RISK OF DEVELOPMENT OF T2D

Tirzepatide treatment exerts concurrent benefits on adiposity and glycaemia, which may prevent progression to T2D and induce normoglycaemia, potentially lessening the risk of CVD among people with prediabetes, as hyperglycaemia is an additional cardiovascular risk factor once diabetes develops.^35^

The 3‐year SURMOUNT‐1 study among adults with prediabetes and obesity or overweight evaluated tirzepatide for long‐term weight management and delay in progression to diabetes.^27^ Over a 176‐week period, tirzepatide treatment was associated with a 94% reduction in the risk of developing T2D compared with placebo (1.2% vs. 12.6%, respectively, for the efficacy estimand; hazard ratio: 0.06; 95% CI: 0.0 to 0.10; *p* < 0.001).^27^ Furthermore, 92% of the tirzepatide‐treated participants with prediabetes had sustained reversion to normoglycaemia. Glycaemic benefits were reported even among tirzepatide‐treated participants who lost less than 5% bodyweight, in keeping with known incretin effects on glycaemia.^27^ These results are consistent with a post hoc risk prediction modelling study demonstrating that tirzepatide treatment was associated with reduced 10‐year predicted risk of developing T2D (either among people with prediabetes at baseline or regardless of prediabetes status at baseline).^36^

## 5 EFFECTS ON CARDIOVASCULAR SAFETY AND MACE AMONG PEOPLE WITH T2D

Considerable evidence of the effects of tirzepatide in terms of improved lipid levels, SBP, DBP, HbA1c and bodyweight comes from clinical trials for T2D, summarized in Table S1 and Figure 1. Additionally, in SURPASS‐4, tirzepatide treatment demonstrated safety in terms of adjudicated MACE‐4 events (cardiovascular death, myocardial infarction, stroke, hospitalization for unstable angina) relative to insulin glargine (hazard ratio: 0.74, 95% CI: 0.51 to 1.08) at 104 weeks.^37^ Furthermore, a pre‐specified meta‐analysis of 7 SURPASS randomized controlled trials found that tirzepatide treatment did not increase the risk of MACE relative to controls.^38^ The overall hazard ratios comparing tirzepatide (at an estimated average dose of 9 mg per week) with a range of different controls (placebo, insulin degludec, insulin glargine, semaglutide 1 mg) for MACE‐4, cardiovascular death and all‐cause death were 0.80 (95% CI: 0.57 to 1.11; *p* = 0.183), 0.90 (95% CI: 0.50 to 1.61) and 0.80 (95% CI: 0.51 to 1.25), respectively.^38^ Cumulatively, these findings support a potential association between tirzepatide and improved cardiovascular safety, though outcomes trials will report soon to give the best evidence.

## 6 TIRZEPATIDE AND HEART FAILURE

The impact of tirzepatide in terms of improved bodyweight reduction, glycaemic control and blood pressure reduction is likely to translate to protective effects against heart failure. These effects were examined in the SUMMIT trial, a phase 3, placebo‐controlled, randomized clinical trial evaluating the safety and efficacy of tirzepatide in adults with heart failure with preserved ejection fraction and obesity, with or without T2D.^39^ Tirzepatide demonstrated a 38% reduction (95% CI: 5% to 59%; *p* = 0.026) in the relative risk of time‐to‐first occurrence of adjudicated death from cardiovascular causes or a worsening heart failure event compared with placebo, though event numbers were modest.^39^ Tirzepatide‐treated participants also reported improved health status and exercise tolerance and decreased high‐sensitivity C‐reactive protein levels relative to placebo (−38.8% vs. −5.9%, respectively; between‐group difference: −34.9%; 95% CI: −45.6 to −22.2; *p* < 0.001).^39^ Moreover, the pre‐specified meta‐analysis of 7 SURPASS trials demonstrated that tirzepatide had a similar point estimate for the risk of hospitalization for heart failure (hazard ratio: 0.67, 95% CI: 0.26 to 1.70) as the SUMMIT trial, accepting wide CIs due to smaller total event numbers.^38^

## 7 SUMMARY OF SAFETY DATA THUS FAR

Tirzepatide use is associated with gastrointestinal symptoms, most commonly nausea, diarrhoea, vomiting, constipation, abdominal pain and dyspepsia, among other adverse effects.^6^ Results from the SURMOUNT and SURPASS trials indicate that the gastrointestinal symptoms following tirzepatide treatment were typically transient and of mild‐to‐moderate severity, with the majority of adverse events occurring at treatment initiation and dose escalation. The frequencies of serious adverse events between tirzepatide‐ and placebo‐treated participants were generally comparable. Furthermore, the SURMOUNT‐1 3‐year study reported decreased incidence of gastrointestinal adverse events with tirzepatide use over time, supporting the long‐term use of tirzepatide among people with obesity or overweight, with or without T2D.^27^ A meta‐analysis of 12 randomized controlled trials of tirzepatide demonstrated that the total incidence of adverse events following tirzepatide treatment was similar to GLP‐1 receptor agonists (dulaglutide and semaglutide) and expectedly higher than placebo and insulin groups.^40^

Among adverse events of special interest, clinical trials have assessed pancreatitis, cholecystitis, hypoglycaemia, MACE and neoplasms (including medullary thyroid cancer). According to a meta‐analysis of 12 randomized clinical trials, the risk of pancreatitis, cholecystitis, MACE‐4 and neoplasms following tirzepatide treatment appears to be comparable to GLP‐1 receptor agonists, placebo or insulin. Tirzepatide is associated with significantly lower hypoglycaemic risk compared with insulin (*p* < 0.01).^40^

## 8 DISCUSSION AND FUTURE DIRECTIONS

Accumulating evidence shows that treatment with tirzepatide confers sustained (up to 3 years) and clinically meaningful weight reduction, alongside improvements in cardiometabolic parameters (including lipids, blood pressure and markers of kidney function), improvements in body composition and liver fat, a significant reduction in the progression to T2D among people with prediabetes and a reduction in the incidence of heart failure events, all of which offer a degree of optimism for the potential cardiovascular benefits of tirzepatide (Figure 1). We recognize the limitation that this article is not a systematic review. Rather, the work is a narrative review of the headline cardiometabolic results reported to date with tirzepatide. We also recognize this article does not discuss mechanisms and pathways by which tirzepatide mediates its actions for benefit. SURPASS‐CVOT (NCT04255433)^20^ and SURMOUNT‐MMO (NCT05556512)^21^ are phase 3 trials evaluating the effects of tirzepatide on the prevention of major cardiovascular events among people with T2D and morbidity and mortality in adults living with obesity, respectively. These ongoing clinical trials will address the gap in hard evidence of the potential cardioprotective benefits of tirzepatide.

## AUTHOR CONTRIBUTIONS

*Design*: Naveed Sattar, Luis‐Emilio García‐Pérez and Emily R. Hankosky. *Conduct/data collection/interpretation*: Naveed Sattar, Luis‐Emilio García‐Pérez, Angel Rodríguez, Richa Kapoor, Adam Stefanski and Emily R. Hankosky. *Writing/critical review of manuscript*: Naveed Sattar, Luis‐Emilio García‐Pérez, Angel Rodríguez, Richa Kapoor, Adam Stefanski and Emily R. Hankosky.

## FUNDING INFORMATION

Eli Lilly and Company, Indianapolis, USA, sponsored this narrative review article.

## CONFLICT OF INTEREST STATEMENT

Naveed Sattar has consulted for and/or received speaker honoraria from Abbott Laboratories, AbbVie, Amgen, AstraZeneca, Boehringer Ingelheim, Eli Lilly, Hanmi Pharmaceuticals, Janssen, Menarini‐Ricerche, Novartis, Novo Nordisk, Pfizer, Roche Diagnostics and Sanofi and grant funding paid to his university from AstraZeneca, Boehringer Ingelheim, Novartis and Roche Diagnostics. Luis‐Emilio García‐Pérez, Angel Rodríguez, Richa Kapoor, Adam Stefanski and Emily R. Hankosky are employees and stockholders of Eli Lilly and Company, IN, USA.

## PEER REVIEW

The peer review history for this article is available at https://www.webofscience.com/api/gateway/wos/peer‐review/10.1111/dom.16549.

## Supporting information


**Table S1.** Results for key cardiovascular disease risk factors from clinical trials for tirzepatide among people with obesity, type 2 diabetes or both.

## Data Availability

Data sharing not applicable to this article as no datasets were generated or analysed during the current study.
