# Supplementary material for: Tirzepatide and cardiometabolic parameters in obesity: Summary of current evidence
Source: Diabetes Obes Metab. 2025 Jun 24;27(10):5386–92. doi: 10.1111/dom.16549 (PMC12409218; doi:10.1111/dom.16549)
Supplement: Supplementary file 1 — Table S1. Results for key cardiovascular disease risk factors from clinical trials for tirzepatide among people with obesity, type 2 diabetes or both. [file DOM-27-5386-s001.docx]

#

# **Supplementary Data**

## **Supplementary Table 1: Results for key cardiovascular disease risk factors from clinical trials for tirzepatide among people with obesity, type 2 diabetes, or both**

| **Study** | **Study population** | **Study arms** | **Study duration (weeks)** | **Change in HbA1c (%)** | **Change in bodyweight (%)** | **Change in SBP (mmHg)** | **Change in DBP (mmHg)** | **Change in LDL-C (%)** | **Change in triglycerides (%)** | **Change in HDL-C (%)** | **Change in waist circumference (cm)** |
| --- | --- | --- | --- | --- | --- | --- | --- | --- | --- | --- | --- |
| SURMOUNT-1^13^ | People with obesity/ overweight without T2D (N=2539) | Placebo (n=643) | 72 | -0.1 | −3.1 | -1.2 | −1.0 | -0.9 | −6.3 | 0.2 | −3.4 |
|  |  | Tirzepatide 5 (n=630), 10 (n=636), 15 (n=630) mg |  | -0.4, -0.5, -0.5 | −16.0, −21.4, −22.5  ETD for 15 mg: -20.1 | −7.0, -8.2, -7.6, | -5.2, -5.5, −4.6 | -5.3, -6.6, -8.6 | -24.3, -27.0, -31.4 | 7.0, 8.6, 8.2 | −14.6, −19.4, −19.9 |
| SURMOUNT-1 3-year study^27^ | People with obesity/ overweight with prediabetes  (N=1032) | Placebo (n=270) | 176 | -0.1 | −2.1 | 0.7 | -1.8 | 1.5 | -4.2 | 2.5 | -2.5 |
|  |  | Tirzepatide 5 (n=247), 10 (n=262), 15 (n=253) mg |  | -0.5, -0.6, -0.7 | −15.4, −19.9, −22.9  ETD for 15 mg: -20.7 | -7.6 (pooled) | -4.8 (pooled) | -7.9 (pooled) | -32.4 (pooled) | 14.1 (pooled) | -12.9, -18.3, -20.0 |
| SURMOUNT-2^16^ | People with obesity/ overweight with T2D (N=938) | Placebo (n=315) | 72 | −0.2 | −3.3 | −1.2 | −0.3 | 6.3 | -5.8 | 1.1 | −3.4 |
|  |  | Tirzepatide 10 (n=312) or 15 (n=311) mg |  | −2.1, −2.2  ETD for 15 mg: -2.1 | −13.4, −15.7  ETD for 15 mg: -12.4 | −5.9, -7.7 | −2.1, -2.9 | 2.3, 3.2  ETD for 15 mg: -3.0 | −26.8, -30.6  ETD for 15 mg: -26.3 | 6.9, 9.6  ETD for 15 mg: 8.4 | −11.2, −13.8  ETD for 15 mg: -10.4 |
| SURMOUNT-3^14^ | People with obesity/ overweight without T2D  (N=579) | Placebo (n=292) | 72 | 0.0 | 3.3 | 4.1 | 2.3 | 6.1 | 3.0 | 3.6 | 0.2 |
|  |  | Tirzepatide MTD (n=287) |  | −0.5  ETD -0.5 | −21.1  ETD -24.5 | −5.1  ETD -9.2 | −3.2  ETD -5.5 | −6.1  ETD -11.5 | −25.8  ETD -28.0 | 15.4  ETD 11.4 | **−**14.6  ETD for TRE: ‑14.8 |
| SURMOUNT-4^15^ | People with obesity/ overweight without T2D  (N=670) | Placebo (n=335) | 52 | -0.2 | -9.5 | -2.4 | -1.7 | 2.6 | -15.3 | 9.4 | -9.1 |
|  |  | Tirzepatide MTD (n=335) |  | -0.6  ETD -0.3 | -26.0  ETD -16.4 | -9.3  ETD -6.9 | -5.5  ETD -3.8 | -5.2  ETD -7.6 | -33.3  ETD -21.2 | 12.3  ETD 2.6 | -22.8  ETD -13.6 |
| SURPASS-1^42^ | People with T2D (N=478) | Placebo (n=115) | 40 | 0.04 | -0.7^#^ | −2.0 | −1.4 | −1.6 | 4.7 | −3.8 | -2.0 |
|  |  | Tirzepatide 5 (n=121), 10 (n=121), 15 (n=121) mg |  | -1.9, -1.9, -2.1  ETD for 15 mg: -2.1 | -7.0^#^, -7.8^#^, -9.5^#^  ETD for 15 mg: -8.8# | -4.7, -5.2, -4.7 | -2.9, -3.1, -3.4 | -6.7, -7.6, -12.4  ETD for 15 mg: -11.0 | -18.5, -18.2, -21.0  ETD for 15 mg: -24.6 | 4.8, 3.2, 7.5  ETD for 15 mg: 11.7 | -5.7, -6.9, -7.2 |
| SURPASS-2^43^ | People with T2D (N=1878) | Semaglutide 1 mg (n=469) | 40 | −1.9 | -6.2^#^ | −3.6* | −1.0* | −6.4 | −11.5 | 4.4 | -5.6* |
|  |  | Tirzepatide 5 (n=470), 10 (n=469), 15 (n=470) mg |  | −2.1, −2.4, −2.5  ETD for 15 mg: -0.6 | -7.8^#^, -10.3^#^, -12.4^#^  ETD for 15 mg: -6.2# | −4.8*, −5.3*, −6.5* | −1.9*, −2.5*, −2.9* | −7.7, −5.6, −5.2 | −19.0, −24.1, −24.8 | 6.8, 7.9, 7.1 | -6.9*, -9.6*, -9.9* |
| SURPASS-3^44^ | People with T2D (N=1437) | Insulin degludec (n=360) | 52 | -1.3 | 2.3^#^ | 0.5 | 0.4 | −2.7 | −12.2 | 1.0 |  |
|  |  | Tirzepatide 5 (n=358), 10 (n=360), 15 (n=359) mg |  | -1.9, -2.2, -2.4  ETD for 15 mg: -1.0 | -7.5^#^, -10.7^#^, -12.9^#^  ETD for 15 mg: -15.2# | −4.9, −6.6, −5.5 | −2.0, −2.5, −1.9 | −6.0, −5.7, −6.6 | −15.4, −26.7, −25.2 | 5.5, 10.2, 10.2 | -7.1 to -10.9 |
| SURPASS-4^37^ | People with T2D (N=1995) | Insulin glargine (n=1000) | 52 | −1.4 | 1.9^#^ | 1.3 | 0.7 | 1.4 | −6.4 | 2.9 | LSM: 110.3 |
|  |  | Tirzepatide 5 (n=329), 10 (n=328), 15 (n=338) mg |  | −2.2, −2.4, −2.6  ETD for 15 mg: -1.1 | -7.1^#^, -9.5^#^, -11.7^#^  ETD for 15 mg: -13.5^#^ | −2.8, −3.7, −4.8 | −1.0, −0.8, −1.0 | −6.8, −8.3, −7.9 | −16.3, −20.1, −22.5 | 6.7, 9.7, 10.8 | LSM: 101.0, 99.1, 98.9 |
| SURPASS-5^45^ | People with T2D (N=475) | Placebo (n=120) | 40 | −0.9 | 1.7^#^ | −1.7* | −2.1* | 2.8 | −6.8 | 1.7 | 1.0 |
|  |  | Tirzepatide 5 (n=116), 10 (n=119), 15 (n=120) mg |  | −2.2, −2.6, −2.6  ETD for 15 mg: -1.7 | -6.2^#^, -8.2^#^, -10.9^#^  ETD for 15 mg: -12.6^#^ | −6.1*, −8.3*, −12.6* | −2.0*, −3.3*, −4.5* | −8.9, −12.8, −15.5 | −15.2, −19.3, −24.9 | 2.1, 1.8, 0.9 | -3.8, -7.4, -8.9 |

The table presents change from baseline (%) and ETD relative to comparators. Results are presented for the efficacy estimand, unless otherwise indicated.

BW, body weight; DBP, diastolic blood pressure; ETD, estimated treatment difference; HbA1c, glycated hemoglobin; HDL-C, high-density lipoprotein cholesterol; LDL-C, low-density lipoprotein cholesterol; LSM: least squares mean; MTD, maximum tolerated dose; SBP, systolic blood pressure; T2D, type 2 diabetes; TRE, treatment-regimen estimand.

* treatment-regimen estimand values

# change in bodyweight reported in kg
